# High‐Energy Topological Edge States and Strain‐Induced Multiple Flat Bands in a Honeycomb Lattice

**DOI:** 10.1002/nap2.70161

**Published:** 2026-06-08

**Authors:** Xiaoqin Bai, Haozhen Tian, Boris A. Malomed, Rongcao Yang, Xiaojun Jia

**Affiliations:** ^1^ School of Physics and Electronics Engineering Shanxi University Taiyuan China; ^2^ Shanxi Key Laboratory of Wireless Communication and Detection Shanxi University Taiyuan China; ^3^ Department of Physical Electronics, School of Electrical Engineering, Faculty of Engineering Tel Aviv University Tel Aviv Israel; ^4^ Instituto de Alta Investigación, Universidad de Tarapacá Arica Chile; ^5^ State Key Laboratory of Quantum Optics Technologies and Devices Institute of Opto‐Electronics, Shanxi University Taiyuan China

**Keywords:** anti‐twig edges, high‐energy edge states, pseudo‐topological edge, strain‐induced multiple flat bands, topologically protected edge

## Abstract

We propose a novel anti‐twig edge in the honeycomb lattice (HCL) that supports two symmetric high‐energy edge states. It is different from the twig edge supporting the zero‐energy flat band. Moreover, multiple flat bands are produced by applying a strain to the HCL with a twig edge or an anti‐twig edge, and the suppression or enhancement of the high‐energy edge state is observed. Under the edge‐parallel stretch strain, the high‐energy edge state band merges into the bulk band, suppressing the high‐energy edge state, whereas the zero‐energy edge state becomes delocalized. On the other hand, under the action of the edge‐parallel compression strain, both the high‐energy and zero‐energy edge states exhibit strong localization. Pseudo‐topological protection of the high‐energy edge state is explored too. Finally, by reconstructing the anti‐twig edge in the HCL, degenerate flat bands and strain‐induced multiple flat bands are produced, and *topologically protected* vacated anti‐twig edge states are demonstrated.

## Graphical abstract

This work proposes a novel anti‐twig edge in the honeycomb lattice that supports two symmetric high‐energy edge states. Moreover, the suppression or enhancement of the high‐energy edge state is observed, and strain‐induced multiple flat bands are produced. By reconstructing anti‐twig edge, degenerate flat bands and topologically protected anti‐twig edge state are demonstrated.

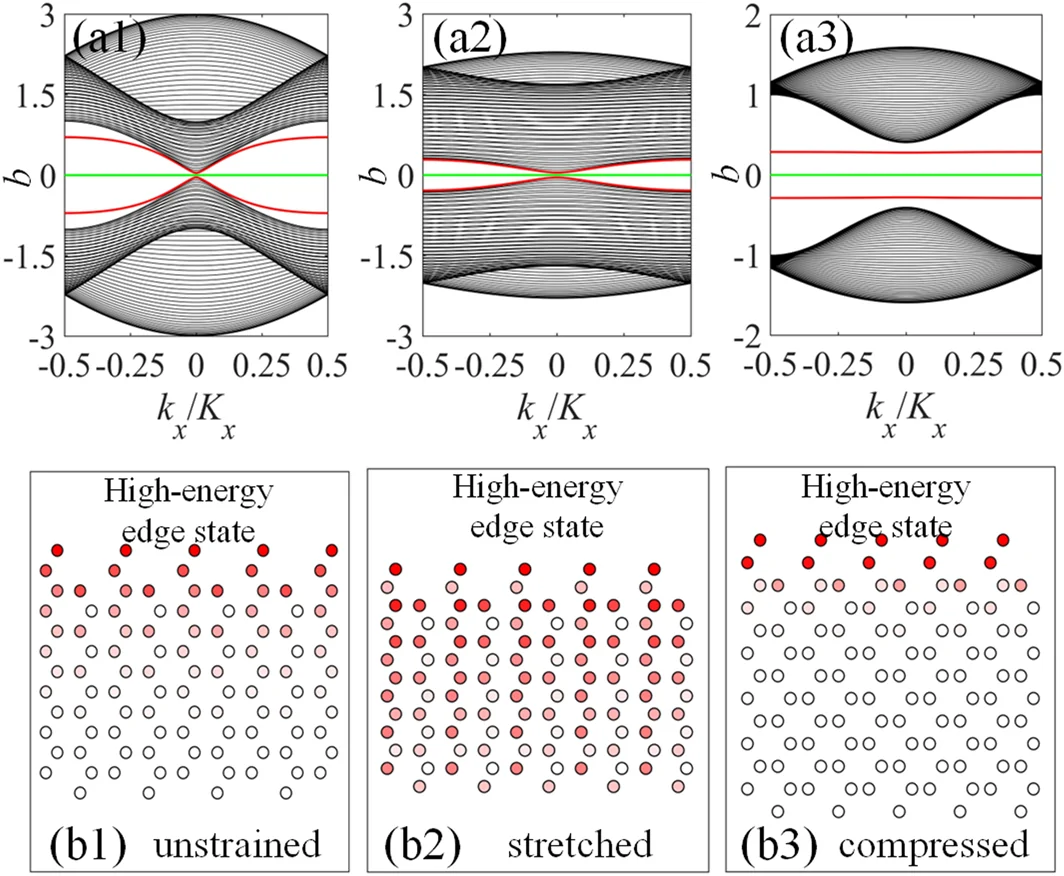

## 1 Introduction

Edge states, which can be topologically nontrivial or trivial ones, are widely supported by various lattices. Among them, topologically protected edge states give rise to a unique phenomenon: the conductance remains confined to the material's surface whereas the bulk remains completely insulating [1, 2]. Topological insulators (TIs), a novel state of matter originally introduced in solid‐state physics, also exhibit this property, hosting the conductance at the surface, while being an insulator in the bulk. This unique characteristic originates from the existence of topological edge states and renders the topological insulator a subject of much interest in studies of acoustics [3, 4], ultracold atoms [5], quantum information [6], and photonics [7, 8, 9, 10]. Photonic TIs are typically constructed using spatially periodic artificial materials, with intrinsic structures such as Lieb [11, 12, 13], kagome [11, 14], honeycomb lattices [9, 15, 16, 17], and others. Among them, the honeycomb lattice (HCL) serves as a versatile platform for the exploration of topological photonics, which can be tailored to feature zigzag, bearded or armchair edges [18]. These edge geometries are known to support edge states. In particular, the zigzag and bearded edges support topological edge states in the first Brillouin zone (BZ), which are nearly complementary with respect to each other, whereas the defect‐free or isotropic armchair edge does not give rise to topological edge states [18]. The zigzag, bearded, and anisotropic armchair edge states have been observed in photonic graphene [18, 19, 20]. In 2023, a distinctive edge configuration named the twig edge was proposed, as one complementary to the armchair edge, by engineering an appropriate primitive cell. The twig edge states are characterized by a topologically protected flat band that spans the entire BZ [21].

More recently, the possibility of the formation of topological edge states, and the existence and dynamics of edge‐state solitons in the presence of nonlinearity have become the focus in studies of topological effects in HCL photonic systems. Topological edge states and a variety of solitons, including dark, bright, vector, and Bragg solitons, have been generated and investigated in spatial inversion‐symmetry‐broken and time‐reversal‐symmetry‐ broken photonic HCLs [22, 23, 24, 25, 26, 27, 28]. Dynamical phenomena in edge states, such as Rabi oscillations [29], Bloch oscillations, and Zener tunneling [17, 30] have also been reported in photonic HCLs.

Applying a strain to photonic lattices can induce a pseudomagnetic field and the corresponding Landau levels [31, 32]. Also, it has been reported that the strain can restore the *PT*‐symmetry in complex photonic HCLs, induce deformations in the energy band, unify valley and anomalous Hall effects, and facilitate the creation and destruction of zigzag and bearded edge states [31, 32, 33, 34, 35]. In strained photonic HCLs with zigzag and bearded terminations, zero‐energy edge states, as well as bright and dark zero‐energy edge solitons, have been demonstrated [36]. Additionally, the inhibition and reconstruction of the Zener tunneling have also been realized in strained photonic HCLs with these terminations [30]. Up to date, despite the growing interest in HCLs, their counterparts with a novel twig edge remained unexplored—in particular, new high‐energy edge states and multiple flat bands are still undisclosed.

In this work, a novel anti‐twig edge in the HCLs is designed, and two symmetric high‐energy edge states are obtained. Moreover, multiple flat bands are produced by applying strains to the configured HCLs, and suppression and enhancement of localization of the high‐energy edge state are explored under the edge‐parallel stretch and compression strains. The localization and pseudo‐topological protection of the high‐energy edge states are demonstrated too. Furthermore, by reconstructing the configured HCLs, degenerate flat band, multiple flat bands, and topological edge states are investigated, along with the associated topological protection.

## 2 Band Structure and Edge States Produced by the Tight‐Binding Approximation

We consider an HCL configuration featuring a specially designed novel edge of the twig type (referred to as an *anti‐twig edge*) along the x direction. In contrast to the twig edge, the anti‐twig one is formed by incorporating an additional lattice site, thus creating a new type of the boundary feature with a reversed twig structure. The HCL with the anti‐twig edge is achieved by selecting the unit cell enclosed by the blue box in Figure 1a, rather than cutting the bulk along the x or y direction [18]. The considered HCL configuration adopts periodic boundary conditions in the x direction with period $L_{x}=3a$ (a is the lattice constant) and open boundary conditions in the *y* direction. Consequently, the Bloch momentum $k_{x}\in \left[-K_{x}/2,K_{x}/2\right]$ ($K_{x}\equiv 2\pi /L_{x}$) is a well‐defined quantum number, unlike $k_{y}$. We choose the super unit cell marked by the shaded rectangle, and calculate the corresponding Bloch Hamiltonian:

(1) $$H=\left[\begin{array}{cccccccc} 0 & H_{12} & 0 & 0 & 0 & 0 & 0 & 0\\ H_{12}^{*} & 0 & 0 & 0 & H_{34} & 0 & 0 & 0\\ 0 & 0 & 0 & H_{34} & H_{35} & 0 & 0 & 0\\ 0 & 0 & H_{34}^{*} & 0 & 0 & H_{35}^{*} & H_{12} & 0\\ 0 & H_{34}^{*} & H_{35}^{*} & 0 & 0 & H_{12} & 0 & 0\\ 0 & 0 & 0 & H_{35} & H_{12}^{*} & 0 & 0 & 0\\ 0 & 0 & 0 & H_{12}^{*} & 0 & 0 & 0 & H_{34}\\ 0 & 0 & 0 & 0 & 0 & 0 & H_{34}^{*} & 0 \end{array}\right],$$

**FIGURE 1:**
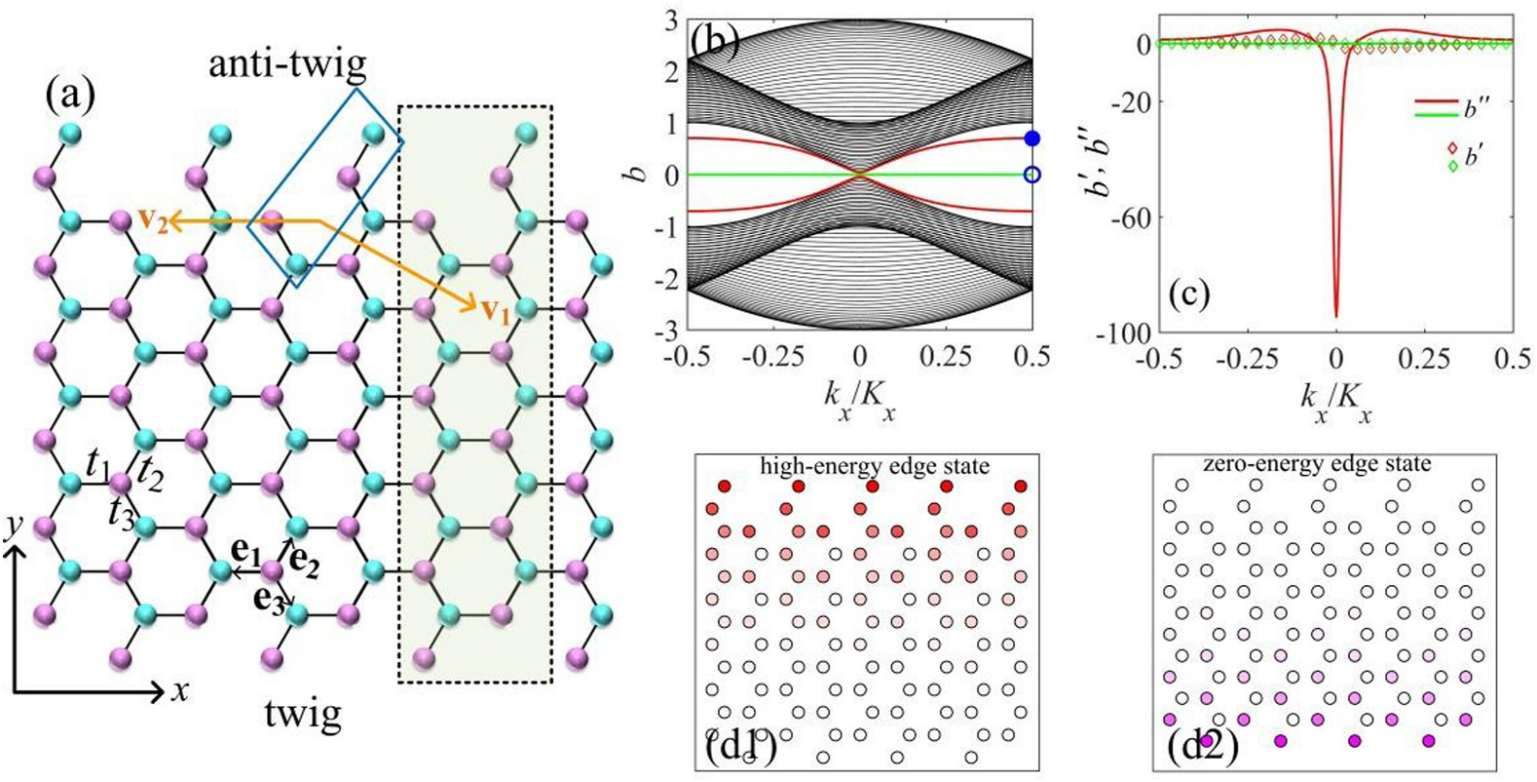
(a) Schematic of the HCL with the anti‐twig (top) and twig (bottom) edges. $t_{1,2,3}$ represents the coupling strength between the nearest‐neighbor sites along three different directions. $e_{1}=\left[-a,0\right]$, $e_{2}=\left[a/2,\sqrt{3}a/2\right]$, and $e_{3}=\left[a/2,-\sqrt{3}a/2\right]$ are bond vectors. The unit cell enclosed by the blue box is defined for anti‐twig edge. $v_{1}=\left[3a,-\sqrt{3}a\right]$ and $v_{2}=\left[-3a,0\right]$ are the Bravais vectors. The shaded rectangle denotes the super unit cell for the construction of Hamiltonian (1). (b) Band structure corresponding to (a), where the red lines represent two symmetric high‐energy edge states and the green line denotes the zero‐energy edge state. (c) Velocities $b^{\prime }$ (hollow rhombuses) and diffraction $b^{\prime \prime }$ (curves) for the upper red branch and green branch of the bands in (b). (d) High‐energy (d1) and zero‐energy (d2) edge states at $k_{x}/K_{x}=0.5$, which correspond to the blue solid dot and hollow dot in (b), respectively.

where $H_{12}=t_{2}\;\exp\left(-ik_{\text{edge}}\cdot e_{2}\right)$, $H_{34}=t_{3}\;\exp\left(ik_{\text{edge}}\cdot e_{3}\right)$, and $H_{35}=t_{1}\;\exp\left(ik_{\text{edge}}\cdot e_{1}\right)$, with $k_{\text{edge}}=\left[k_{x},0\right]$ being the Bloch wave vector, $t_{1,2,3}$ are strengths of the coupling between the nearest‐neighbor sites along three different directions, defined by bond vectors $e_{1}=\left[-a,0\right]$, $e_{2}=\left[a/2,\sqrt{3}a/2\right]$, and $e_{3}=\left[a/2,-\sqrt{3}a/2\right]$. The corresponding band structure for $t_{1}=t_{2}=t_{3}=1$ and a=1.4 is plotted in Figure 1b. One can clearly see that the proposed HCL supports three edge bands, which are marked by two mutually symmetric red lines and the horizontal green one. The red and green lines are associated with the symmetric edge states which belong, respectively, to the anti‐twig and twig edges [see examples of the edge states in Figure 1d1,d2]. Note that the second‐order derivative, that is, the diffraction, of the anti‐twig edge states corresponding to the red bands is nonzero [as indicated by the red curve in Figure 1c]; therefore, we designate the corresponding edge state as high‐energy edge state; on the contrary, the green band remains completely flat [see the green line in Figure 1c], suggesting to designate the corresponding mode as a zero‐energy edge state.

We consider an example displayed in Figure 1d with a specific Bloch wave vector $k_{x}/K_{x}=0.5$, which supports strong localization of the high‐energy and zero‐energy edge states. We stress that a decrease of the Bloch wavenumber leads to expansion of both the high‐energy and zero‐energy edge states into the bulk, as shown in Figure 2, which compares the localization of the edge states for $k_{x}/K_{x}=0.5$ and $k_{x}/K_{x}=0.3$. It is seen that reducing the Bloch wavenumber $k_{x}$ narrows the separation between the edge bands and the bulk band [see Figure 2a1,a2], resulting in weaker localization of both the high‐energy and zero‐energy edge states, as seen in Figure 2b1,b2, respectively. Moreover, it is evident that the high‐energy and zero‐energy edge states exhibit exponential localization at their respective anti‐twig and twig edges, underscoring the distinct behavior of these edge states.

**FIGURE 2:**
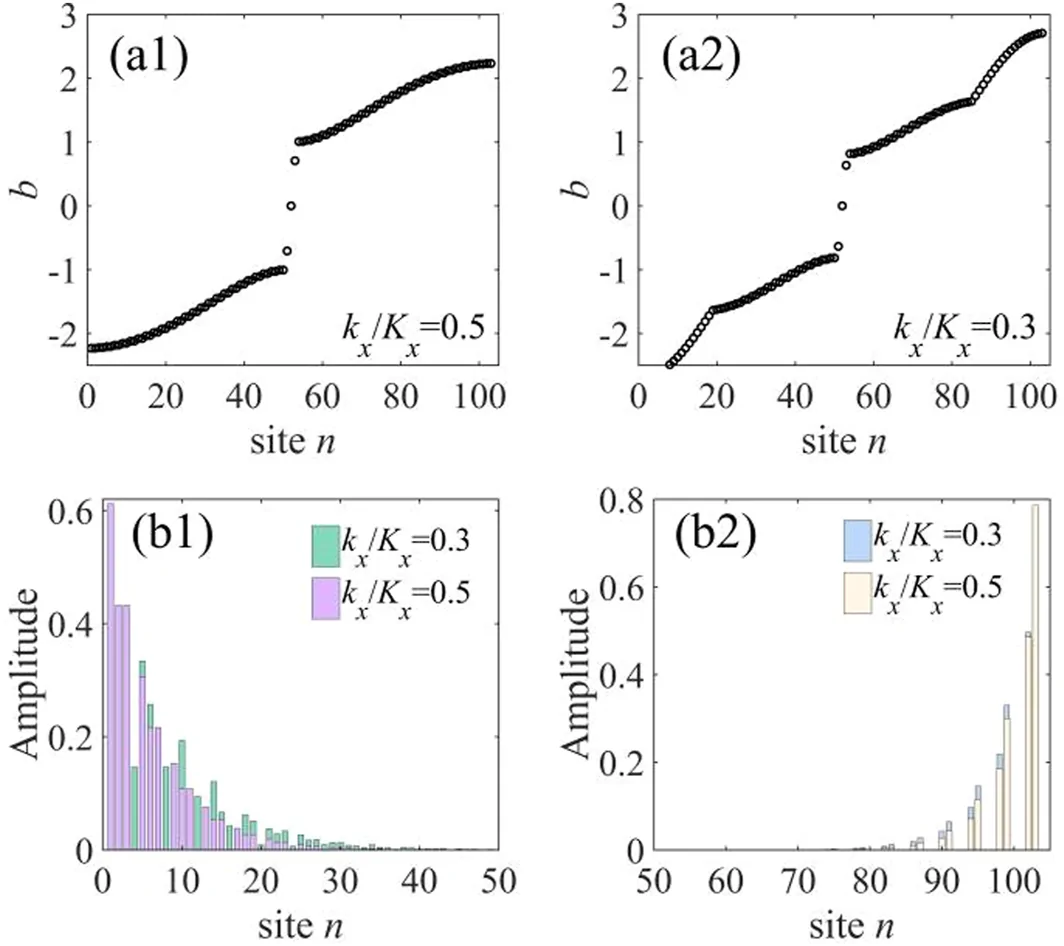
(a) Eigenvalue spectra for $k_{x}/K_{x}=0.5$ (a1) and $k_{x}/K_{x}=0.3$ (a2) corresponding to Figure 1b. Panels (b1) and (b2) display, respectively, the high‐energy and zero‐energy edge states, for $k_{x}/K_{x}=0.5$ and $k_{x}/K_{x}=0.3$.

## 3 Optical Realization of the Proposed HCL

In this section, we introduce a feasible physical realization of the proposed HCL, in which the lattice sites can be defined in terms of the refractive‐index variation. The photonic lattice can be regarded as an array of waveguides arranged in a honeycomb structure. The desired coupling strength can be established by adjusting the spatial separation between the waveguides. In the photonic HCL, the propagation of light along the z axis is governed by the Schrödinger‐like paraxial wave equation for the dimensionless light field Ψ:

(2) $$i\frac{\partial \Psi }{\partial z}=-\frac{1}{2}\left(\frac{\partial^{2}}{\partial x^{2}}+\frac{\partial^{2}}{\partial y^{2}}\right)\Psi -R\left(x,y\right)\Psi ,$$

where x and y are the normalized transverse coordinates, z is the propagation distance. $R\left(x,y\right)=p\sum_{m,n}\exp\left[-{\left(x-x_{m}\right)}^{2}/d_{x}^{2}-{\left(y-y_{n}\right)}^{2}/d_{y}^{2}\right]$ describes the profiles of the waveguide array as the combination of Gaussian functions, where $\left(x_{m},y_{n}\right)$ denotes the positions of the waveguides, $d_{x}$ and $d_{y}$ represent the width of a single waveguide, and p is the depth. Such photonic HCL can be fabricated using the femtosecond laser writing method [15, 36]. We choose the representative parameters as p=10, $d_{x}=d_{y}=0.5$, and a=1.4, which, respectively, correspond to the refractive‐index modulation depth $∼10^{-3}$, waveguide width 5μm, and lattice constant 14μm, assuming that laser radiation is coupled into the waveguide at the wavelength of 800 nm, with a characteristic transverse size of 10 μm. Accordingly, the lattice with the anti‐twig and twig edges, displayed in Figure 1a, can be emulated optically using the waveguide arrays represented by the potential term ∼ R(x,y), as shown in Figure 3a. In these waveguide arrays, we investigate the propagation of the high‐energy and zero‐energy edge states through direct simulation using Equation (2), and employ an elongated Gaussian excitation condition $\Psi \left(x,y,z=0\right)=A_{0}\;\exp\left[-x^{2}/w_{x}^{2}-{\left(y-y_{0}\right)}^{2}/w_{y}^{2}\right]$ with $A_{0}$ being the amplitude of the excitation, $w_{x}$ and $w_{y}$ denoting the width of the excitation, and $y_{0}$ representing the position of the edges along the y axis. The corresponding propagation dynamics of the high‐energy and zero‐energy edge states for $k_{x}/K_{x}=0.5$ and $k_{x}/K_{x}=0.3$ are shown in Figure 3b,c. One can easily find that the beams initially break up into the patterns that corresponding to edge states illustrated in Figure 1d1,d2. Subsequently, the beams propagate along the edges without obvious scatter into bulk for $k_{x}/K_{x}=0.5$, as shown in Figure 3b1,b2. However, for $k_{x}/K_{x}=0.3$, the reduced localization of both the high‐energy and zero‐energy edge states implies the weak penetration into bulk, as depicted in Figure 3c1,c2. Moreover, it can be observed by comparing Figure 3c1 with Figure 3b1 that the high‐energy edge state at $k_{x}/K_{x}=0.3$ experiences significant diffraction, which is attributed to the non‐zero second‐order derivative.

**FIGURE 3:**
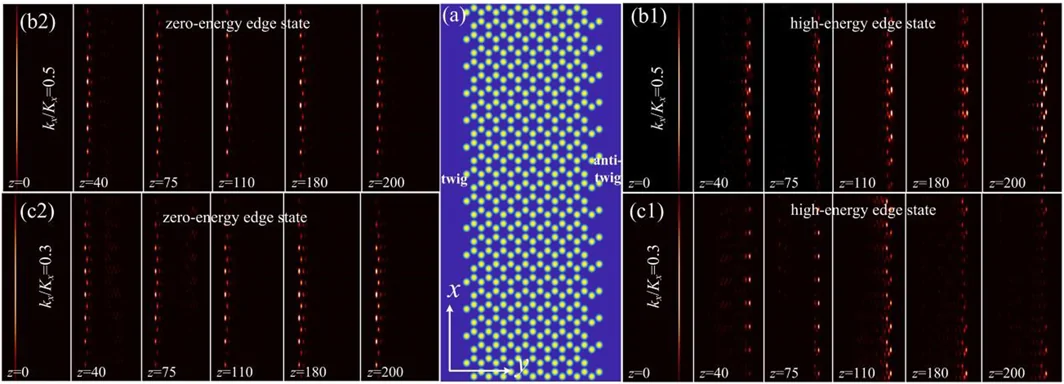
(a) Optical realization of the HCL with anti‐twig and twig edges. (b, c) Propagation of the high‐energy (b1, c1) and zero‐energy edge states (b2, c2) at $k_{x}/K_{x}=0.5$ (b) and $k_{x}/K_{x}=0.3$ (c). Other parameters: $A_{0}=1$, $w_{x}=20a/\sqrt{3}$, $w_{y}=0.5a/\sqrt{3}$.

## 4 Strained Photonic HCL

By applying a strain to the proposed HCL, the coupling strength between sites can be efficiently controlled, which can further lead to the deformation of the energy bands. For the transversely stretched HCL (applying an edge‐parallel stretch strain), the nearest‐neighbor distance between horizontal sites is stretched and the coupling strength is reduced [see Figure 4a], that is, $\left|e_{1}\right|>\left|e_{2,3}\right|$ and $t_{1}<t_{2,3}$. In this case, the transversely stretched HCL can be looked upon as a stack of trivial dimer Su‐Schrieffer‐ Heeger (SSH) chains with stronger intra‐cell hopping, as shaded by the gray‐dashed region in Figure 4a, thereby, the edge state is inherently not supported. Figure 4b illustrates the band structures of the transversely stretched HCL. It is seen that the upper and lower edge bands tend to merge with the bulk band, and the middle flat band approaches the bulk band, as shown in Figure 4b1. The decreasing ratio of coupling strength $\delta_{s}=t_{1}/t_{2}$ promotes the eventual disappearance of the upper and lower symmetric edge bands and a narrowing of the separation between the flat band and the bulk band, as presented in Figure 4b2. This suggests that for the transversely stretched HCL, the modes exhibit notably delocalization, concentrating in both the bulk and edges, as shown in Figure 4c1,c2. The wavefunction of the modes shown in Figure 4d further indicates the delocalization and the substantial penetration into the bulk. The underlying mechanism for these phenomena is that the stretch strain increases the separation between transverse sites, thereby suppressing the coupling strength. Concurrently, the stretching reduces the lattice confinement, which pushes the high‐energy edge band into the bulk. As a result, the energy of the edge states eventually merges with the bulk as the stretch strain intensifies.

**FIGURE 4:**
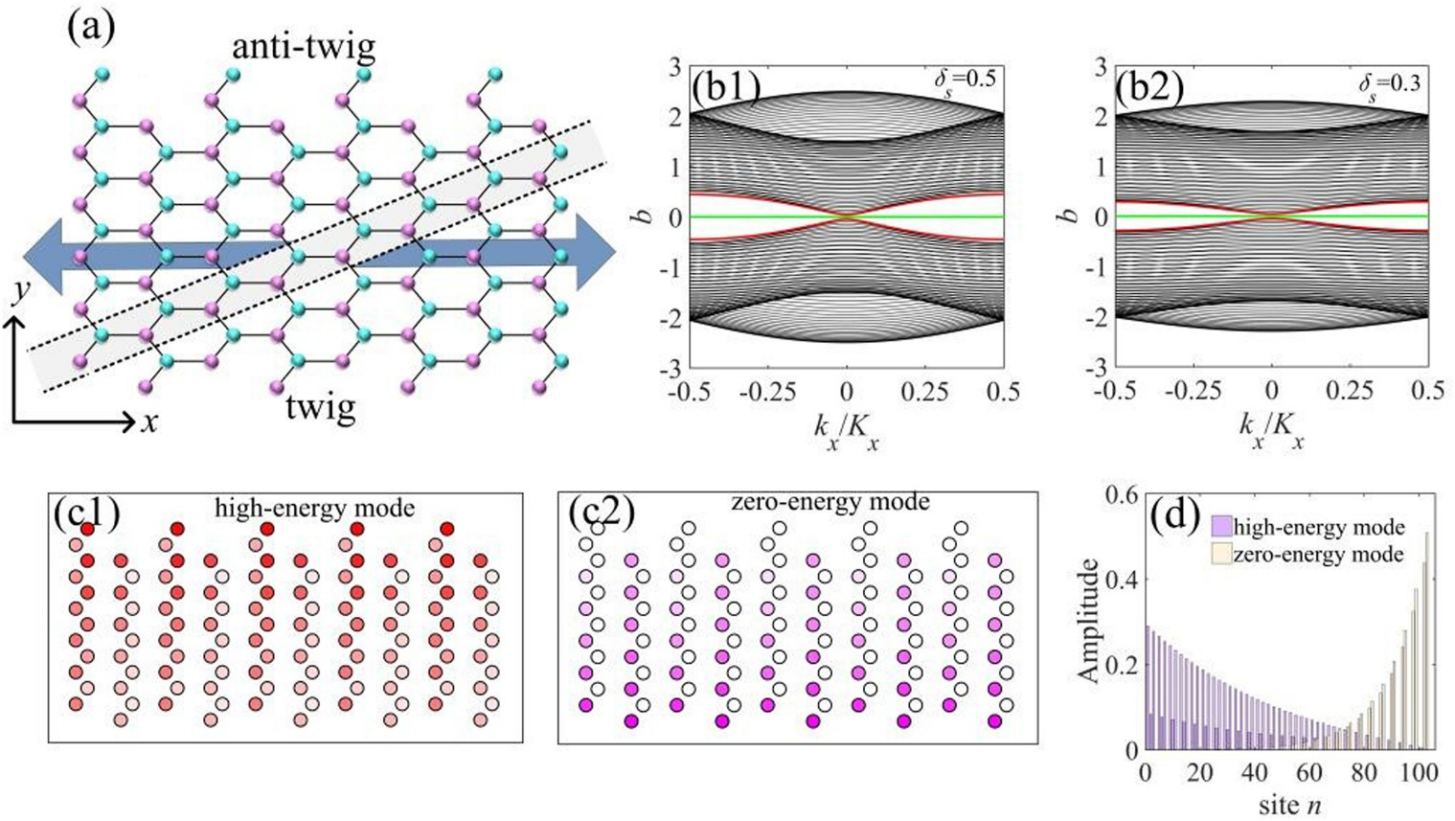
(a) Schematic of the transversely stretched HCL with anti‐twig and twig edges. Blue arrows represent the direction of stretch strain. (b) Band structures of the transversely stretched HCL corresponding to (a) for $\delta_{s}=0.5$ (b1) and $\delta_{s}=0.3$ (b2). (c) High‐energy (c1) and zero‐energy (c2) modes for $k_{x}/K_{x}=0.5$. (d) Wavefunction of the high‐energy and zero‐energy modes corresponding to (c).

For the transversely compressed HCL (applying an edge‐parallel compression strain), the nearest‐neighbor horizontal distance between sites is compressed, that is, $\left|e_{1}\right|<\left|e_{2,3}\right|$ and $t_{1}>t_{2,3}$. In this case, the strained HCL can be regarded as a stack of SSH chains with weaker intra‐cell coupling [36], as highlighted by the gray shading in Figure 5a. Figure 5b1,b2 show the band structures for different ratios of coupling strength $\delta_{c}=t_{2}/t_{1}$. It can be seen that decreasing the ratio $\delta_{c}$ leads to the disappearance of Dirac point, the opening of band gap, and the emergence of multiple flat bands. This indicates the strong localization of the higher‐energy and zero‐energy edge states, as shown in Figure 5c1,c2,d. These phenomena occur because compression strain decreases the transverse site distance and enhances the hopping integral and coupling strength. Also, the strain‐induced anisotropy opens the bandgap, flattens the energy bands, producing flat bands characterized by highly localized states with zero group velocity. It is noted that here the obtained additional non‐degenerate edge states are entirely different from that reported in Ref. [37] in which the strained graphene lattice possesses degenerate flat bands.

**FIGURE 5:**
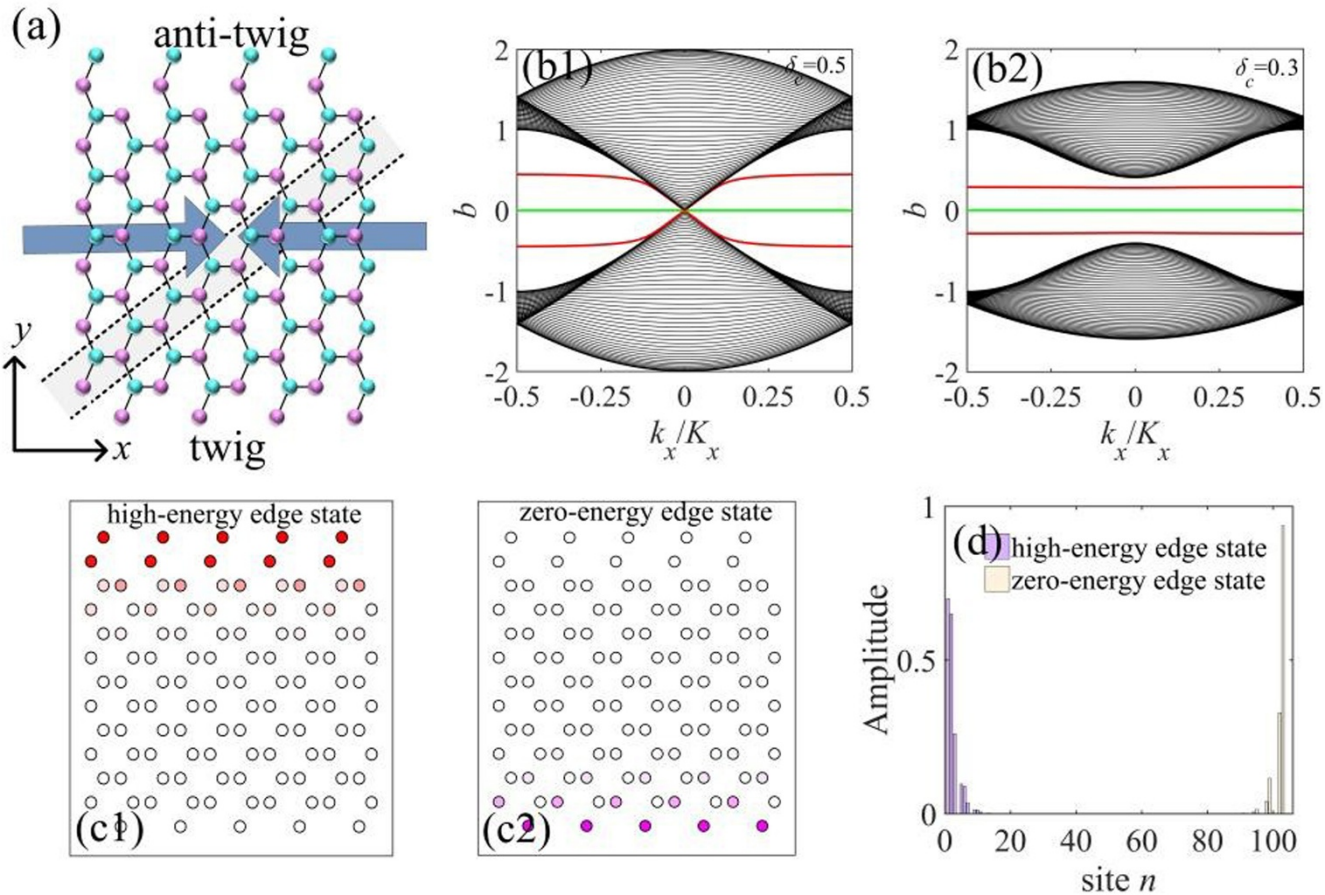
(a) Schematic of the transversely compressed HCL with anti‐twig and twig edges. Blue arrows represent the direction of compression strain. (b) Band structures of the transversely compressed HCL corresponding to (a) for $\delta_{c}=0.5$ (b1) and $\delta_{c}=0.3$ (b2). (c) High‐energy (c1) and zero‐energy (c2) edge states for $k_{x}/K_{x}=0.5$. (d) Wavefunction of the high‐energy and zero‐energy edge states corresponding to (c).

To seek conditions for the opening of a bandgap and the emergence of multiple flat bands, we first construct the bulk Hamiltonian by selecting the blue‐box unit cell in Figure 1a, based on the relation between the anti‐twig edge and the bulk. The bulk Hamiltonian is

(3) $$H=\left[\begin{array}{cc} 0 & M_{k}\\ M_{k}^{*} & 0 \end{array}\right],$$

where $M_{k}$ is 2×2 matrix,

(4) $$M_{k}=\left[\begin{array}{cc} t_{2}+t_{3}e^{-ik_{\text{bulk}}\cdot \left(v_{1}+v_{2}\right)} & t_{1}e^{-ik_{\text{bulk}}\cdot \left(v_{1}+2v_{2}\right)}\\ t_{1}e^{ik_{\text{bulk}}\cdot \left(v_{1}+v_{2}\right)} & t_{3}+t_{2}e^{ik_{\text{bulk}}\cdot \left(v_{1}+v_{2}\right)} \end{array}\right],$$

with the Bloch wave vector $k_{\text{bulk}}=\left[k_{x},k_{y}\right]$ and the Bravais vectors $v_{1}=\left[3a,-\sqrt{3}a\right]$, $v_{2}=\left[-3a,0\right]$. Diagonalizing the bulk Hamiltonian (3), we obtain the eigenvalues

(5) $$b_{1∼4}\left(k_{x},k_{y}\right)=\pm {\left[t_{1}^{2}+4t_{2}^{2}\;\cos^{2}\left(\frac{\sqrt{3}}{2}ak_{y}\right)\pm 4t_{1}t_{2}\;\cos\left(\frac{\sqrt{3}}{2}ak_{y}\right)\cos\left(\frac{3}{2}ak_{x}\right)\right]}^{\frac{1}{2}},$$

which represents the band structure of the proposed HCL. The bandgap closes when $b_{2,3}=0$ at $k_{x}=0$. Thus, the condition for the bandgap opening is

(6) $$\frac{t_{1}}{2t_{2}}=\cos\left(\frac{\sqrt{3}}{2}ak_{y}\right).$$

Equation (6) indicates that the bandgap always closes when $t_{1}<2t_{2}$ for $-\pi /\sqrt{3}a<\left|k_{y}\right|<\pi /\sqrt{3}a$. In other words, when $t_{1}>2t_{2}$, the Dirac point disappears and the bandgap reopens. The band structures are shown in Figures 1, 4 and 5b, following the above derivation.

In the optically mimicked lattice, the strained HCL configuration can be realized by precisely manipulating the spatial separation of the waveguides. Figure 6a,c, respectively, present the continuous transversely stretched and compressed HCLs corresponding to the configurations depicted in Figures 4a and 5a. In these continuous strained HCLs, the propagation dynamics of the high‐energy state and zero‐energy state at $k_{x}/K_{x}=0.5$ are shown in Figure 6b,d. The results in Figure 6b reveal that in the transversely stretched lattice, both the high‐energy mode and zero‐energy mode exhibit significant spreading into the bulk during propagating. The characteristic is consistent with that described in Figure 4c,d. In contrast, in the transversely compressed lattice, both the high‐energy and zero‐energy edge states consistently maintain perfect localization at the anti‐twig and twig edges during propagating, being characterized by the absence of diffraction, backscattering, and energy leakage into bulk, as depicted in Figure 6d.

**FIGURE 6:**
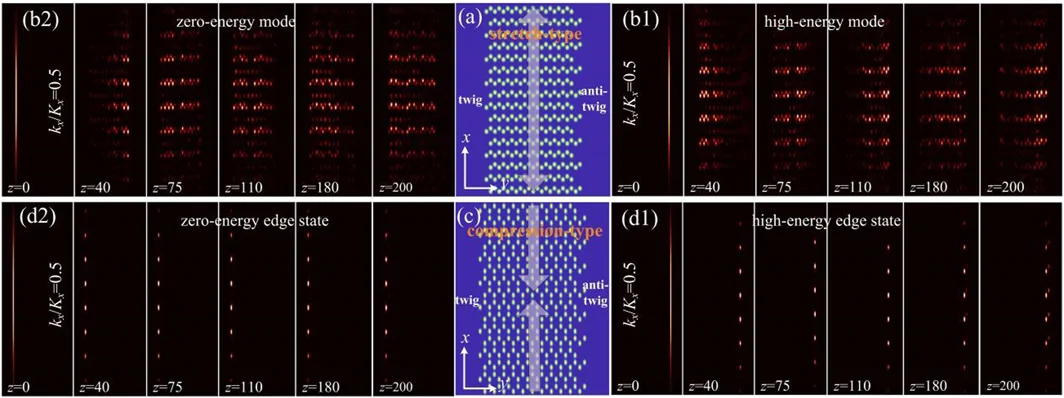
(a, c) Optical transversely stretched and compressed HCLs. Purple arrows represent the direction of the strain. (b, d) Propagation of the high‐energy state (b1, d1) and zero‐energy state (b2, d2) at $k_{x}/K_{x}=0.5$ in the transversely stretched (b) and compressed (d) HCLs. Other parameters are the same as in Figure 3.

## 5 The Pseudo‐Topological Nature of the Edge States

To explore the topological properties and robust localization of the high‐energy edge state, we calculate the winding number as suggested by works [21, 37, 38, 39]:

(7) $$w=-\frac{1}{2\pi }\int_{0}^{2\pi }\frac{d}{dk}\arg\left(\det\left(M_{k}\right)\right)dk,$$

in which k is $k_{x}$ or $k_{y}$, depending on the edge direction. To calculate the winding number for the anti‐twig edge states, the Bloch wave vector $k_{x}$ is fixed, and the integration is carried out along $k_{y}$ [37, 38, 39]. The resulting winding loops of the high‐energy edge state are plotted in the $\left(\sigma_{x},\sigma_{y}\right)$ plane in Figure 7a1–a3. For comparison, the winding loops of the zero‐energy edge state [21, 37] are also illustrated in Figure 7a. The origin *O*, marked by a green dot, is encircled by the winding loops of the zero‐energy edge state [see the blue loops in Figure 7a1–a3], indicating a nonzero winding number and thus its topologically nontrivial nature in the unstrained transversely stretched and compressed HCLs. The red lines in the figures suggest that the high‐energy edge states at the anti‐twig edge are topologically trivial. However, strong localization of the high‐energy edge states at the anti‐twig edge is manifested even in a defective HCL shown in Figure 7b. The propagation dynamics of the high‐energy and zero‐energy edge states in the transversely compressed lattice with defects are displayed in Figure 7c. It can be observed that the high‐energy edge state localizes strongly at the lattice edges with defects in the course of the propagation, resembling the topological protection behavior of the zero‐energy edge state. Therefore, we characterize such localization behavior of the high‐energy edge state as *pseudo‐topological protection*. Furthermore, it is noteworthy that the persistent localization of the edge states near the defects is attributed to zero velocity of the edge states at $k_{x}/K_{x}=0.5$.

**FIGURE 7:**
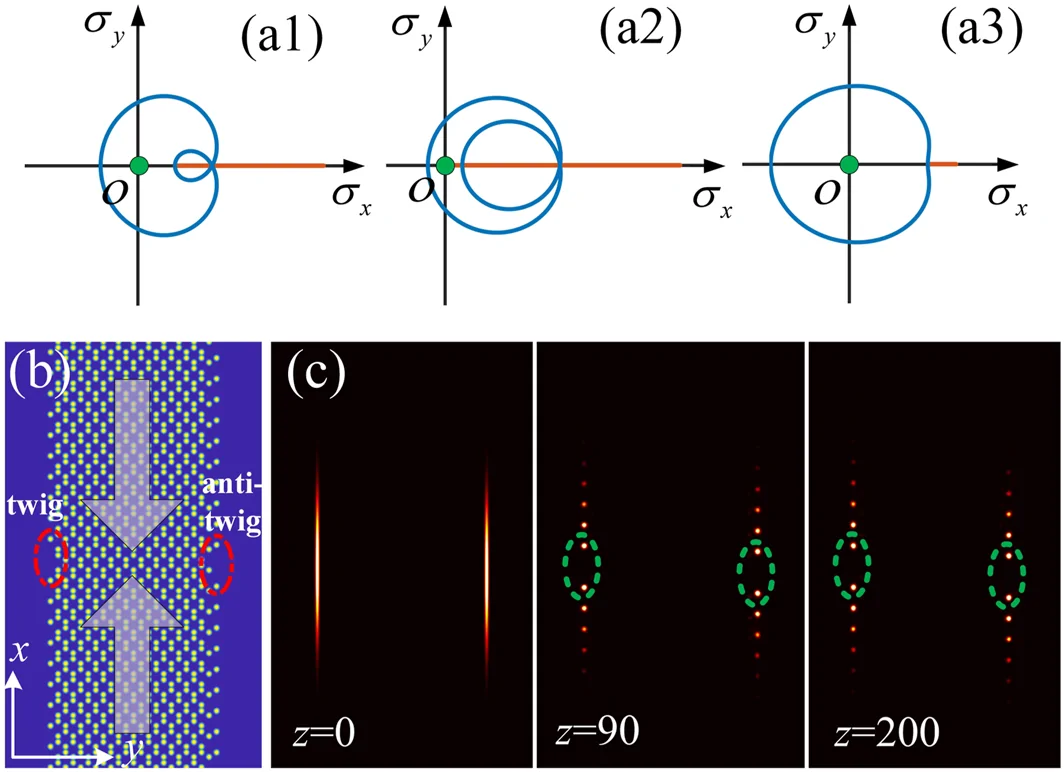
(a) Winding loops of the high‐energy (red lines) and zero‐energy (blue curves) edge states for $k_{x}/K_{x}=0.5$ in unstrained (a1), transversely stretched (a2), and compressed (a3) HCLs. (b) A transversely compressed HCL with two respective defects at anti‐twig and twig edges, marked by the red circles. (c) Propagation of the edge states in the transversely compressed lattice shown in (b).

## 6 Topologically Protected Vacated Anti‐Twig Edge States

To produce true topological edge states, we reconstruct the anti‐twig edge pattern by removing the sites enclosed by the dashed circles in Figure 8a. The so reconstructed anti‐twig edge is referred to as a *vacated anti‐twig edge*, to distinguish it from the anti‐twig edge discussed above. In contrast to the pseudo‐topological anti‐twig edge shown in Figure 1a, the vacated anti‐twig edge is a *topologically protected one*, as demonstrated below. The band structures of the unstrained, transversely stretched, and compressed HCLs with a vacated anti‐twig edge are shown in Figure 8b1–b3, and the corresponding eigenvalue spectra for $k_{x}/K_{x}=0.5$ are displayed in Figure 8c1–c3. It is seen that flat bands at the edges appear and the transverse compression strain also leads to multiple flat bands. Note that, unlike the cases of the pseudo‐topological anti‐twig edge shown in Figures 1, 4 and 5b, degenerate flat bands are observed, as marked in Figure 8c1–c3. Moreover, the energy of the degenerate edge states at the vacated anti‐twig edge is localized on the blue sublattices, as depicted in Figure 9a. Sharp exponential localization is clearly observed in both the unstrained and transversely compressed HCLs [Figure 9a1,a3], in contrast to the weak localization in the stretched lattice [Figure 9a2].

**FIGURE 8:**
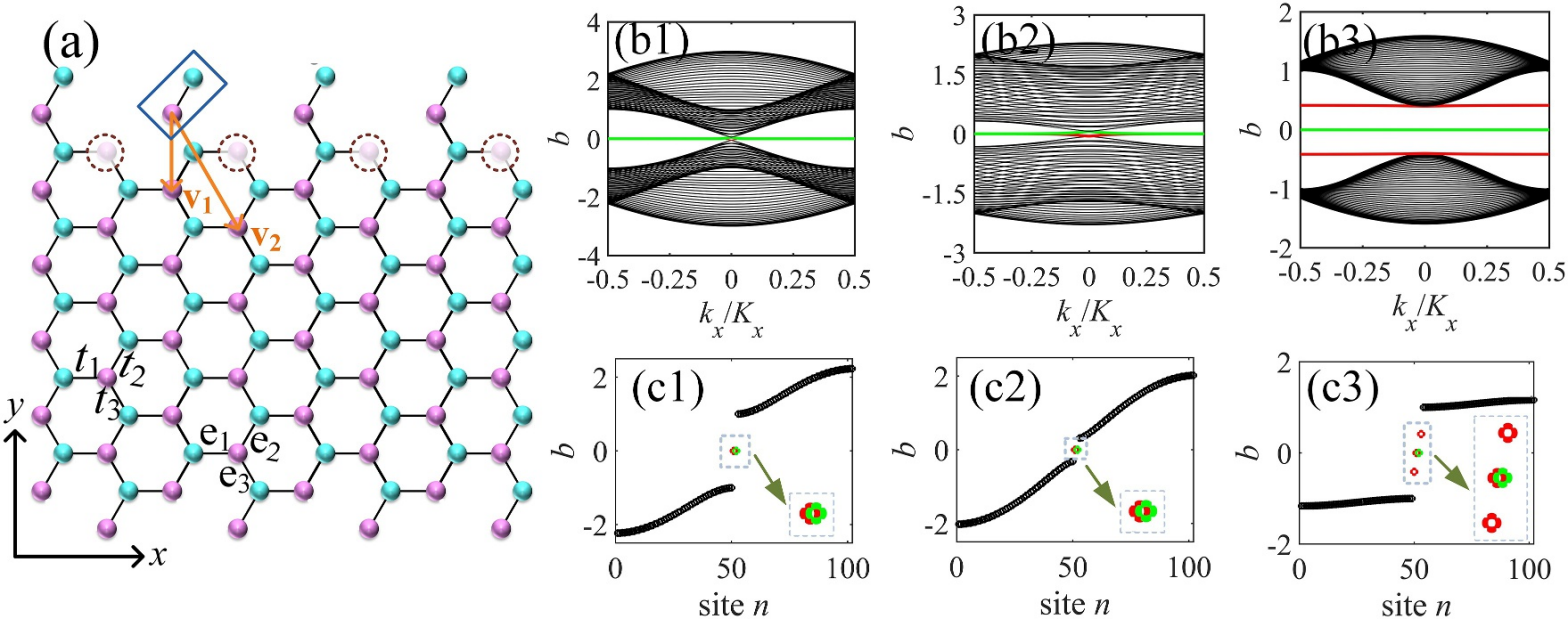
(a) Schematic of the HCL with the topologically protected vacated anti‐twig (top) and twig (bottom) edges. The unit cell enclosed by the blue box pertains to the vacated anti‐twig edge. $v_{1}=\left[0,-\sqrt{3}a\right]$ and $v_{2}=\left[3a/2,-3\sqrt{3}a/2\right]$ are the Bravais vectors. (b) Band structures of unstrained (b1), transversely stretched (b2), and compressed (b3) HCLs, where the middle lines represent two degenerate flat bands. (c) Eigenvalue spectra for $k_{x}/K_{x}=0.5$ corresponding to (b).

**FIGURE 9:**
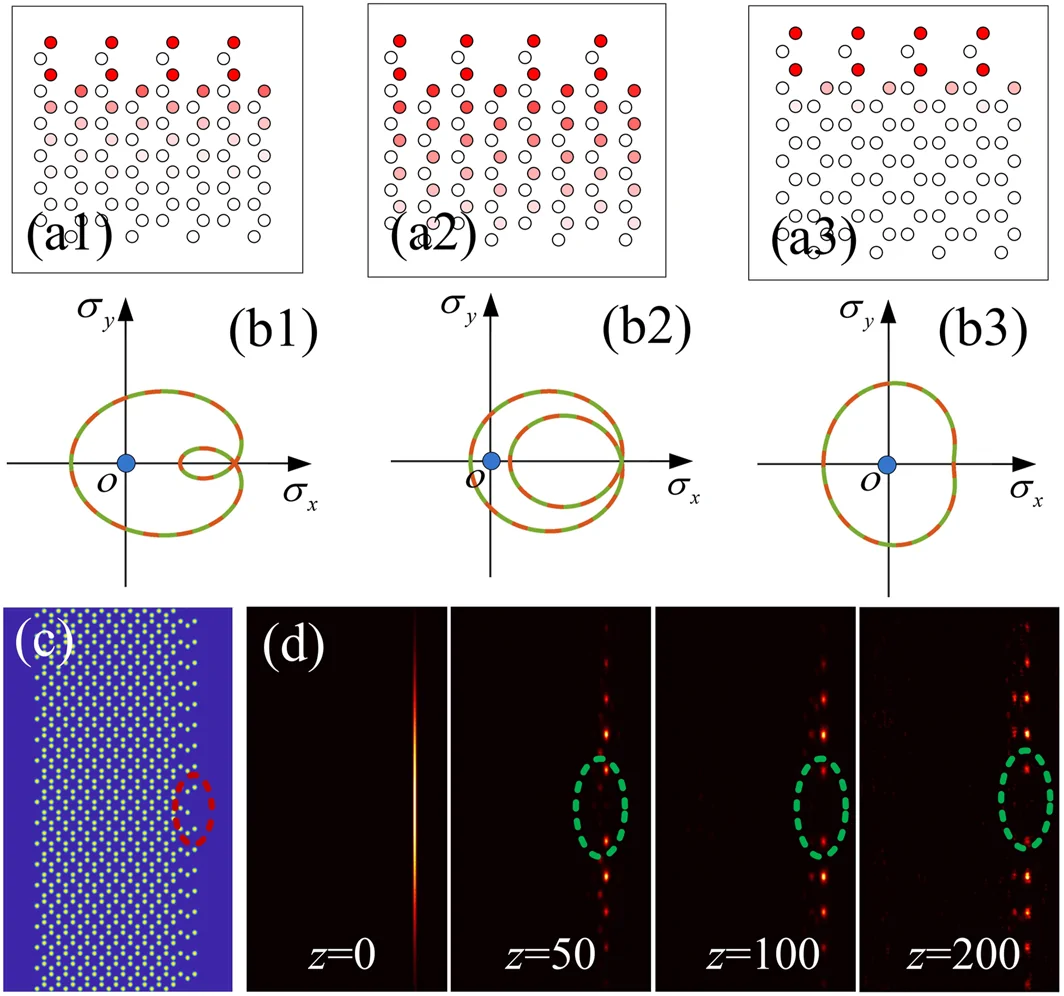
(a) Degenerate edge states at the topologically protected vacated anti‐twig edge in unstrained (a1), transversely stretched (a2), and compressed (a3) HCLs. (b) Winding loops at the vacated anti‐twig edge (red solid curves) and the twig edge (green dashed curves) for $k_{x}/K_{x}=0.5$ in unstrained (b1), transversely stretched (b2), and compressed (b3) HCLs. (c) Transversely compressed HCL with a defect (red circle) at the vacated anti‐twig edge. (d) Propagation of the edge state at the edge shown in (c).

To demonstrate the topological nature of the edge states, the unit cell, marked by the blue box in Figure 8a, is chosen to describe the reconstructed HCL with the vacated anti‐twig edge. The corresponding bulk Hamiltonian is written as

(8) $$H=\left[\begin{array}{cc} 0 & Q_{k}\\ Q_{k}^{*} & 0 \end{array}\right],Q_{k}=t_{2}+t_{1}e^{ik\cdot \left(-2v_{1}+v_{2}\right)}+t_{3}e^{-ik\cdot v_{1}},$$

where $k=\left[k_{x},k_{y}\right]$ is the Bloch wave vector and $v_{1}=\left[0,-\sqrt{3}a\right]$ and $v_{2}=\left[3a/2,-3\sqrt{3}a/2\right]$ are Bravais vectors. The winding loops of the edge states at the vacated anti‐twig edge are calculated as per Equations (7) and (8). As shown in Figure 9b, the circle around the origin *O* (marked by the blue dots) indicates that the edge states are topologically nontrivial according to the bulk‐boundary correspondence, with winding loops identical to those at the twig edge. Furthermore, the topological protection of the edge states is examined in the transversely compressed HCL with a defect at the vacated anti‐twig edge [see Figure 9c]. Figure 9d displays the propagation of the edge state, which passes the defect without diffraction or backscattering, confirming the topological protection of the anti‐twig edge state.

## 7 Conclusion

In summary, we have investigated the high‐energy edge state in the HCL with a novel anti‐twig edge. It is found that the localization of the high‐energy edge state depends on the Bloch momenta. By applying strains, we demonstrated the suppression and enhancement of localization of both the high‐energy and zero‐energy edge states in the HCL. In the transversely stretched HCL, the bands of high‐energy and zero‐energy edge states undergo progressive disappearance and significant coupling into bulk, respectively, resulting in the delocalization of these states. In the transversely compressed HCL, multiple flat bands appear, and both the high‐energy and zero‐energy edge states exhibit profound localization at the edges. Employing an optical continuous model, the propagation dynamics of the high‐energy and zero‐energy edge states are simulated. The topological property of the high‐energy edge state is explored by winding loops, and the pseudo‐topological protection of the edge state is observed in the transversely compressed HCL. Furthermore, degenerate flat bands and strain‐induced multiple flat bands are achieved through reconstructing the anti‐twig edge into its topologically protected counterpart. The topological nature and protection of the corresponding edge states are demonstrated. Our research may expand the geometrical construction of topological insulators and underscore the distinct responses to mechanical strain across different lattice types.

## Author Contributions

**Xiaoqin Bai:** writing – original draft, methodology, formal analysis, data curation. **Haozhen Tian:** writing – review and editing, software. **Boris A. Malomed:** conceptualization, formal analysis, writing – review and editing. **Rongcao Yang:** supervision, resources, methodology, funding acquisition, conceptualization. **Xiaojun Jia:** conceptualization, resources, supervision.

## Funding

This work was supported by the National Natural Science Foundation of China (Grant Nos. 62575165, 62305199), the Natural Science Foundation of Shanxi Province (Grant No. 202203021221016) and Shanxi graduate Education Innovation Program (Grant No. 2024YZ06).

## Conflicts of Interest

The authors declare no conflicts of interest.

## Data Availability

The data that supports the findings of this study are available from the corresponding author upon reasonable request.

## References

[1] M. Z. Hasan and C. L. Kane , “Topological Insulators,” Reviews of Modern Physics 82, no. 4 (2010): 3045–3067, 10.1103/revmodphys.82.3045.

[2] X. L. Qi and S. C. Zhang , “Topological Insulators and Superconductors,” Reviews of Modern Physics 83, no. 4 (2011): 1057–1110, 10.1103/revmodphys.83.1057.

[3] H. R. Xue , Y. H. Yang , and B. L. Zhang , “Topological Acoustics,” Nature Reviews Materials 7, no. 2 (2022): 974–990, 10.1038/s41578-022-00465-6.

[4] B. L. Zhang , “Topological Acoustics Unlocks New Horizons in Microfluidics,” Nature Materials 24, no. 5 (2025): 652–653, 10.1038/s41563-025-02136-7. 40119035

[5] C. Braun , R. Saint‐Jalm , A. Hesse , J. Arceri , I. Bloch , and M. Aidelsburger , “Real‐Space Detection and Manipulation of Topological Edge Modes With Ultracold Atoms,” Nature Physics 20, no. 8 (2024): 1306–1312, 10.1038/s41567-024-02506-z.

[6] F. M. D’Angelis , F. A. Pinheiro , D. Guéry‐Odelin , S. Longhi , and F. Impens , “Fast and Robust Quantum State Transfer in a Topological su‐Schrieffer‐Heeger Chain With Next‐to‐Nearest‐Neighbor Interactions,” Physical Review Research 2, no. 3 (2020): 033475, 10.1103/physrevresearch.2.033475.

[7] A. Blanco‐Redondo , I. Andonegui , M. J. Collins , et al., “Topological Optical Waveguiding in Silicon and the Transition Between Topological and Trivial Defect States,” Physical Review Letters 116, no. 16 (2016): 163901, 10.1103/physrevlett.116.163901. 27152805

[8] J. F. Li , Y. Wang , Z. X. Zhou , et al., “Experimental Observations of Communication in Blackout, Topological Waveguiding and Dirac Zero‐Index Property in Plasma Sheath,” Nanophotonics 12, no. 10 (2023): 1847–1856, 10.1515/nanoph-2022-0800. PMC11614341 39635142

[9] A. Kumar , Y. J. Tan , N. Navaratna , M. Gupta , P. Pitchappa , and R. Singh , “Slow Light Topological Photonics With Counter‐Propagating Waves and Its Active Control on a Chip,” Nature Communications 15, no. 1 (2024): 926, 10.1038/s41467-024-45175-5. PMC10830473 38296983

[10] Y. V. Kartashov , “Quadratic Solitons in Higher‐Order Topological Insulators,” Chaos, Solitons & Fractals 194 (2025): 116199, 10.1016/j.chaos.2025.116199.

[11] M. J. Ablowitz and J. T. Cole , “Topological Insulators in Longitudinally Driven Waveguides: Lieb and Kagome Lattices,” Physics Reviews 99, no. 3 (2019): 033821, 10.1103/physreva.99.033821.

[12] C. Y. Li , F. W. Ye , X. F. Chen , et al., “Lieb Polariton Topological Insulators,” Physical Review B: Condensed Matter 97, no. 8 (2018): 081103, 10.1103/physrevb.97.081103.

[13] S. K. Ivanov , Y. V. Kartashov , L. J. Maczewsky , A. Szameit , and V. V. Konotop , “Edge Solitons in Lieb Topological Floquet Insulator,” Optics Letters 45, no. 6 (2020): 1459–1462, 10.1364/ol.385494. 32163991

[14] K. Prabith , G. Theocharis , and R. Chaunsali , “Nonlinear Corner States in a Topologically Nontrivial Kagome Lattice,” Physical Review B: Condensed Matter 110, no. 10 (2025): 104307, 10.1103/physrevb.110.104307.

[15] M. C. Rechtsman , J. M. Zeuner , Y. Plotnik , et al., “Photonic Floquet Topological Insulators,” Nature 496, no. 7444 (2013): 196–200, 10.1038/nature12066. 23579677

[16] X. X. Wu , Y. Meng , J. X. Tian , et al., “Direct Observation of Valley‐Polarized Topological Edge States in Designer Surface Plasmon Crystals,” Nature Communications 8, no. 1 (2017): 1304, 10.1038/s41467-017-01515-2. PMC5670222 29101323

[17] N. Khan , P. Wang , Q. D. Fu , C. Shang , and F. W. Ye , “Observation of Period‐Doubling Bloch Oscillations,” Physical Review Letters 132, no. 5 (2024): 053801, 10.1103/physrevlett.132.053801. 38364161

[18] M. Kohmoto and Y. Hasegawa , “Zero Modes and Edge States of the Honeycomb Lattice,” Physical Review B: Condensed Matter 76, no. 20 (2007): 205402, 10.1103/physrevb.76.205402.

[19] Y. Plotnik , M. C. Rechtsman , D. H. Song , et al., “Observation of Unconventional Edge States in ‘Photonic Graphene’,” Nature Materials 13, no. 1 (2014): 57–62, 10.1038/nmat3783. 24193661

[20] S. T. Song , Y. Teng , W. C. Tang , et al., “Janus Graphene Nanoribbons With Localized States on a Single Zigzag Edge,” Nature 637, no. 8046 (2025): 580–586, 10.1038/s41586-024-08296-x. 39779862

[21] S. Q. Xia , Y. S. Liang , L. Q. Tang , D. H. Song , J. J. Xu , and Z. G. Chen , “Photonic Realization of a Generic Type of Graphene Edge States Exhibiting Topological Flat Band,” Physical Review Letters 131, no. 1 (2023): 013804, 10.1103/physrevlett.131.013804. 37478443

[22] J. Noh , S. Huang , K. P. Chen , and M. C. Rechtsman , “Observation of Photonic Topological Valley Hall Edge States,” Physical Review Letters 120, no. 6 (2018): 063902, 10.1103/physrevlett.120.063902. 29481241

[23] Z. Y. Zhang , R. Wang , Y. Q. Zhang , et al., “Observation of Edge Solitons in Photonic Graphene,” Nature Communications 11, no. 1 (2020): 1902, 10.1038/s41467-020-15635-9. PMC7171198 32312996

[24] S. K. Ivanov , Y. V. Kartashov , L. J. Maczewsky , A. Szameit , and V. V. Konotop , “Bragg Solitons in Topological Floquet Insulators,” Optics Letters 45, no. 8 (2020): 2271–2274, 10.1364/ol.390694. 32287211

[25] S. K. Ivanov and Y. V. Kartashov , “Floquet Valley Hall Edge Solitons,” Chaos, Solitons & Fractals 186 (2024): 115239, 10.1016/j.chaos.2024.115239.

[26] H. Zhong , Y. V. Kartashov , Y. D. Li , M. Li , and Y. Q. Zhang , “Topological Edge States in a Photonic Floquet Insulator With Unpaired Dirac Cones,” Photonics Research 12, no. 10 (2024): 2078–2087, 10.1364/prj.524824.

[27] Q. Tang , Y. Q. Zhang , Y. V. Kartashov , Y. D. Li , and V. V. Konotop , “Vector Valley Hall Edge Solitons in Superhoneycomb Lattices,” Chaos, Solitons & Fractals 161 (2022): 112364, 10.1016/j.chaos.2022.112364.

[28] S. Shen , C. Shang , Y. D. Li , and Y. Q. Zhang , “Chiral Bulk Solitons in Photonic Graphene With Decorated Boundaries,” Laser & Photonics Reviews 19, no. 3 (2025): 2401114, 10.1002/lpor.202401114.

[29] Y. Q. Zhang , Y. V. Kartashov , Y. P. Zhang , L. Torner , and D. V. Skryabin , “Resonant Edge‐State Switching in Polariton Topological Insulators,” Laser & Photonics Reviews 12, no. 8 (2018): 1700348, 10.1002/lpor.201700348.

[30] Y. J. Chang , Y. H. Lu , Y. Y. Yang , et al., “Inhibition and Reconstruction of Zener Tunneling in Photonic Honeycomb Lattices,” Advances in Materials 34, no. 28 (2022): 2110044, 10.1002/adma.202110044. 35306698

[31] M. C. Rechtsman , J. M. Zeuner , A. Tünnermann , S. Nolte , M. Segev , and A. Szameit , “Strain‐Induced Pseudomagnetic Field and Photonic Landau Levels in Dielectric Structures,” Nature Photonics 7, no. 2 (2013): 153–158, 10.1038/nphoton.2012.302.

[32] M. Barsukova , F. Grisé , Z. Y. Zhang , et al., “Direct Observation of Landau Levels in Silicon Photonic Crystals,” Nature Photonics 18, no. 6 (2024): 580–585, 10.1038/s41566-024-01425-y.

[33] A. Szameit , M. C. Rechtsman , O. Bahat‐Treidel , and M. Segev , “PT‐Symmetry in Honeycomb Photonic Lattices,” Physics Reviews 84, no. 2 (2011): 021806, 10.1103/physreva.84.021806.

[34] M. C. Rechtsman , Y. Plotnik , J. M. Zeuner , et al., “Topological Creation and Destruction of Edge States in Photonic Graphene,” Physical Review Letters 111, no. 10 (2013): 103901, 10.1103/physrevlett.111.103901. 25166669

[35] J. L. Yuan , H. Cai , C. J. Wu , S. Y. Zhu , R. B. Liu , and D. W. Wang , “Unification of Valley and Anomalous Hall Effects in a Strained Lattice,” Physical Review B: Condensed Matter 104, no. 3 (2021): 035410, 10.1103/physrevb.104.035410.

[36] B. Q. Ren , H. G. Wang , M. R. Belic , Y. D. Li , X. Y. Zhu , and Y. Q. Zhang , “Zero‐Energy Edge States and Solitons in Strained Photonic Graphene,” Physics Reviews 107, no. 4 (2023): 043504, 10.1103/physreva.107.043504.

[37] Y. S. Liang , J. Y. Zhan , S. Q. Xia , D. H. Song , and Z. G. Chen , “Observation of Doubly Degenerate Topological Flatbands of Edge States in Strained Graphene,” Advanced Photonics 7, no. 4 (2025): 046005, 10.1117/1.AP.7.4.046005.

[38] J. F. Li , J. F. Yao , Y. Wang , Z. X. Zhou , Z. H. Lan , and C. X. Yuan , “Topological States Decorated by Twig Boundary in Plasma Photonic Crystals,” Advanced Optical Materials 12, no. 17 (2024): 2303244, 10.1002/adom.202303244.

[39] S. S. Ryu , A. P. Schnyder , A. Furusaki , and A. W. W. Ludwig , “Topological Insulators and Superconductors: Tenfold Way and Dimensional Hierarchy,” New Journal of Physics 12, no. 6 (2010): 065010, 10.1088/1367-2630/12/6/065010.

